# Identification of MicroRNAs as Diagnostic Biomarkers for Breast Cancer Based on the Cancer Genome Atlas

**DOI:** 10.3390/diagnostics11010107

**Published:** 2021-01-11

**Authors:** Jungho Kim

**Affiliations:** Department of Biomedical Laboratory Science, College of Health Sciences, Catholic University of Pusan, Busan 46252, Korea; jutosa70@cup.ac.kr; Tel.: +82-51-510-0660; Fax: +82-51-510-0568

**Keywords:** breast cancer, microRNAs, diagnosis, TCGA, translational research

## Abstract

Breast cancer is the most common cancer among women worldwide. MicroRNAs (miRNAs or miRs) play an important role in tumorigenesis, and thus, they have been identified as potential targets for translational research with diagnostic, prognostic, and therapeutic markers. This study aimed to identify differentially expressed (DE) miRNAs in breast cancer using the Cancer Genome Atlas. The miRNA profiles of 755 breast cancer tissues and 86 adjacent non-cancerous breast tissues were analyzed using Multi Experiment Viewer; miRNA–mRNA network analyses and constructed KEGG pathways with the predicted target genes were performed. The clinical relevance of miRNAs was investigated using area under the receiver operating characteristic curve (AUC) analysis, sensitivity, and specificity. The analysis identified 28 DE miRNAs in breast cancer tissues, including nine upregulated and 19 downregulated miRNAs, compared to non-cancerous breast tissues (*p* < 0.001). The AUC for each DE miRNA, miR-10b, miR-21, miR-96, miR-99a, miR-100, miR-125b-1, miR-125b-2, miR-139, miR-141, miR-145, miR-182, miR-183, miR-195, miR-200a, miR-337, miR-429, and let-7c, exceeded 0.9, indicating excellent diagnostic performance in breast cancer. Moreover, 1381 potential target genes were predicted using the prediction database tool, miRNet. These genes are related to PD-L1 expression and PD-1 checkpoint in cancer, MAPK signaling, apoptosis, and TNF pathways; hence, they regulate the development, progression, and immune escape of cancer. Thus, these 28 miRNAs can serve as prospective biomarkers for the diagnosis of breast cancer. Taken together, these results provide insight into the pathogenic mechanisms and potential therapies for breast cancer.

## 1. Introduction

Breast cancer is the third most common malignancy among women, with annual morbidity increasing worldwide [1]. According to the World Health Organization, 2.1 million new cases and 627,000 deaths were estimated for breast cancer in 2018. Moreover, breast cancer accounts for approximately 15% of all cancer-related deaths in women [2]. Breast cancer is a heterogeneous disease classified into four subtypes by gene expression profiling, including luminal A (ER/PR+, HER2−, Ki67+ < 20%), luminal B (ER/PR+ < 20%, HER2−, Ki67+ ≥ 20%), HER2 (ER/PR−, HER2 overexpression), and basal-like (ER−, PR−, HER2−) [3,4].

Early detection and improved treatment can aid in better survival and outcomes in patients with breast cancer. Mammography for breast cancer is a widely used screening tool. However, the extensive use of mammography has been hindered by the cost and expertise required for mammography. On the other hand, alternative methods, such as ultrasound screening, are highly operator-dependent. In addition, tumor serum markers, such as carbohydrate antigen 15–3 (CA-15–3) and carcinoembryonic antigen (CEA), are nonspecific and have limited sensitivity and specificity [5,6].

Even though well-characterized subtypes and early detection have reduced the burden of treatment for patients, more specific molecular targets are needed to increase the survival rate for each patient. One of the molecular targets with growing interest is microRNA (miRNA or miR). It has been used to assess the diagnosis, prognosis, and therapy response in breast cancer [7,8,9,10]; miRNAs are small, naturally occurring, non-coding RNAs (18–25 nucleotides) that regulate gene expression, mainly by binding to the 3ʹ untranslated region of target mRNAs. They lead to the silencing of respective gene expression either by direct degradation of mRNA or inhibition of protein translation [11,12]; miRNAs are involved in a wide range of cancer biology processes, including regulation of target mRNA expression, which promotes tumor growth, apoptosis, progression, metastasis, and immune evasion [13,14,15,16]. Many studies have reported that miRNAs play key roles in the occurrence and progression of breast cancer [17,18,19,20]; this has prompted translational studies on miRNAs for breast cancer to be actively conducted [21].

In recent years, the development of high-throughput technologies and bioinformatics analysis has provided new insights into novel cancer biomarkers and therapeutic target information [22,23,24]; miRNAs can regulate the expression of multiple genes rather than one gene, affect the activity of the entire signaling network, and modulate biological processes. Previous studies have estimated that miRNAs can target more than 5300 human genes, constituting over 30% of the human genome [25]. Moreover, comprehensive analysis of miRNAs and target genes can provide new opportunities for the prevention, treatment, and diagnosis of breast cancer. In this study, datasets of miRNAs in 755 breast tumors and 86 adjacent non-tumor breast tissues from the Cancer Genome Atlas (TCGA) were used to identify differentially expressed (DE) miRNAs. The diagnostic utility of DE miRNAs was evaluated in terms of the area under the curve (AUC) of the receiver operating characteristic (ROC), sensitivity, and specificity. DE miRNAs were also investigated according to cancer stage and subtype. Furthermore, the target genes predicted by miRNet were further explored using pathway analysis to determine their potential roles in breast cancer.

## 2. Materials and Methods

### 2.1. The Cancer Genome Atlas (TCGA) Data Analysis

Raw data for miRNAs and clinical information of breast cancer were obtained from the TCGA open source repository (http://firebrowse.org/) on 01/28/2016. To verify clinical diagnostic values, data for all clinical samples, including age, race, tumor stage, molecular subtype, and reads per million miRNAs, were included for 755 breast cancer samples and 86 adjacent non-cancerous breast tissues. Other clinical variables (treatment, surgical type, etc.) were not analyzed in the current study. Data were divided into different stages, including early stage (stages 1 and 2), locally advanced stage (stage 3), and metastatic stage (stage 4). Data from eight samples with unknown stages were excluded from this study. Clinical information from TCGA data is shown in Table S1 (Supplementary Material).

### 2.2. miRNA Expression Profiles

To determine miRNA expression profiles and identify DE miRNAs, hierarchical clustering and volcano plot analyses were performed using Multi Experiment Viewer (MEV) software version 4.4. Principal component analysis (PCA) was also performed to assess population clustering and the parameters responsible for the distinction between the groups. The mean values of DE miRNAs between cancerous and non-cancerous breast tissues were compared using Student’s *t*-test, and the false discovery rate-adjusted *p*-value (*q*-value) was calculated.

### 2.3. Constructin Regulatory Network between miRNAs and Their Targets and Pathway Enrichment Analysis

The target genes of the selected DE miRNAs were predicted using miRNet (http://www.mirnet.ca/). The miRNA target gene network was constructed based on mapping analysis [22].

Furthermore, the target genes in the network were analyzed using Cytoscape software version 3.8.1 with ClueGO for the Kyoto Encyclopedia of Genes and Genomes (KEGG) pathway enrichment analysis. ClueGO parameters were set as indicated: GO term fusion selected; only display pathways with *p* < 0.001 with Bonferroni step-down analysis; and kappa score of 0.4 [23].

### 2.4. Statistical Analysis

All statistical analyses were performed using GraphPad Prism software version 6 (La Jolla, CA, USA), SPSS Statistics software (version 21.0; IBM, Armonk, NY, USA), and Multi Experiment Viewer (MEV) software version 4.4. Student’s *t*-test was used to compare the expression of miRNAs between cancerous and non-cancerous breast tissues. Receiver operating characteristic (ROC) curve analysis and the area under the ROC curve (AUC) were used to assess the diagnostic utility of the selected miRNAs. Analysis of the association between survival and DE miRNAs was performed using miRpower, a web tool to validate survival-associated miRNAs [26]. A database was established using miRNA expression data from the Molecular Taxonomy of Breast Cancer International Consortium (METABRIC). Survival was estimated using Kaplan methods and evaluated using a log-rank test. In all analyses, *p* < 0.05 was considered statistically significant.

## 3. Results

### 3.1. Patients’ Characteristics

The miRNA sequencing dataset comprising a total of 755 breast cancer and 86 adjacent non-cancerous breast tissues was obtained from the TCGA breast cancer project. Demographic data and clinical characteristics of the patients are shown in Table 1. The percentage of female patients was 98.8 % (746/755). The patients were aged 58.42 ± 13.11 years (range, 26–90 years). The most frequent race of patients was white (71.31%), followed by black or African American (20.66%), Asian (7.42), and unknown (0.79). There were 567 patients with early stage cancer (75.10%), 171 with locally advanced stage (22.62%), nine with metastatic stage (1.19%), and eight with unknown (1.06%). Moreover, 208 patients presented with luminal A (27.55%), 74 with luminal B (9.80%), 116 with HER2-positive (15.36%), and 357 with triple-negative breast cancer (TNBC) (47.28%) subtypes.

### 3.2. Selection of 28 Potential miRNAs as Diagnostic Biomarkers for Breast Cancer

**Figure 1:**
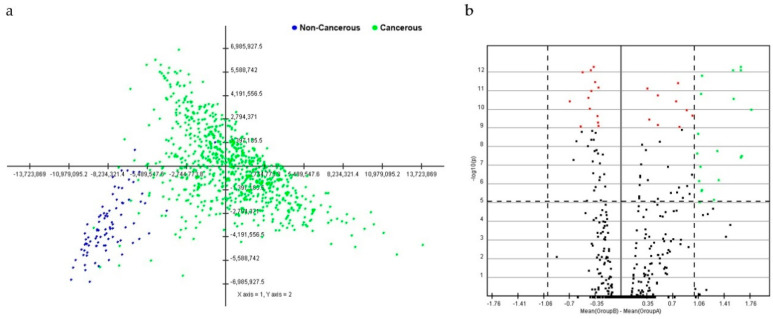
Principal component analysis (PCA) score and volcano plot in breast cancerous and non-cancerous tissues. (**a**) In the score plot, each dot represents an individual, and is colored in accordance with the embedded legend. (**b**) Volcano plot of miRNAs between breast cancerous and non-cancerous tissues. Cutoff points for the *p*-value (<0.00001; −log_10_(0.00001) = 1) or mean difference (>1 or <−1) are indicated with dotted lines.

**Figure 2:**
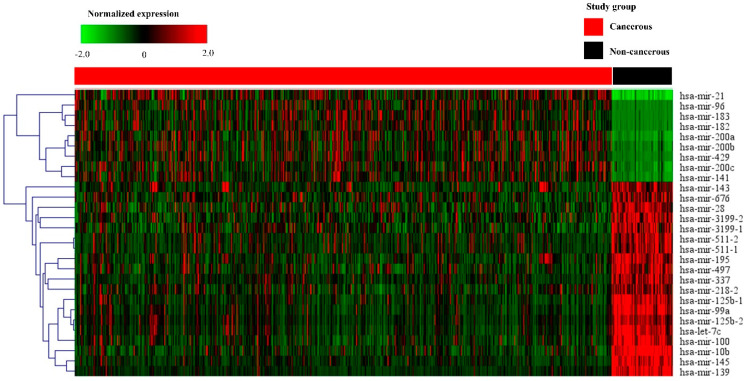
Heat map of the selected 28 miRNAs in cancerous and non-cancerous tissues. The heat map is obtained using the two-way hierarchical clustering of 28 significantly expressed miRNAs (Pearson correlation, *p* < 0.05 by hierarchical clustering analysis). A red dot represents upregulated miRNA, and a green dot represents downregulated miRNA.

PCA with data from breast cancer tissues was distinguished from that of non-cancerous breast tissues. A cluster distinction was generated using PCA (Figure 1a). Furthermore, DE miRNAs were investigated using the *q-*value. Subsequently, more potential DE miRNAs were selected based on the mean difference (>1 or <–1) and –log10 (*p*) < 5; and 28 DE miRNAs, including nine upregulated and 19 downregulated miRNAs, were identified by volcano plot analysis (Figure 1b). The heat map profiles of the expression of the 28 selected miRNAs in breast cancer tissues and non-cancerous breast tissues are shown in Figure 2. These miRNAs, including miR-21, miR-96, miR-141, miR-182, miR-183, miR-200a, miR-200b, miR-200c, and miR-429, which were upregulated, and miR-10b, miR-28, miR-99a, miR-100, miR-125b-1, miR-125b-2, miR-139, miR-143, miR-145, miR-195, miR-218-2, miR-337, miR-497, miR-511-1, miR-511-2, miR-676, miR-3199-1, miR-3199-2, and let-7c were downregulated compared to non-cancerous breast tissues. 

### 3.3. Diagnostic Utility of Selected miRNAs

**Figure 3:**
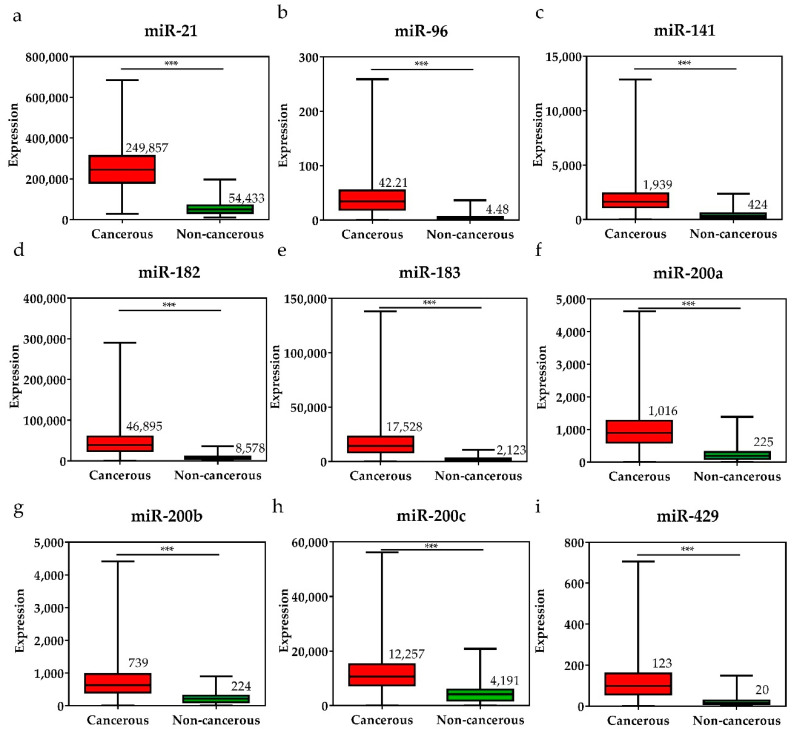
The expression levels of upregulated miRNAs in breast cancer tissues and the pair-matched non-cancerous tissues. The miRNAs upregulated in breast cancer were (**a**) miR-21, (**b**) miR-96, (**c**) miR-141, (**d**) miR-182, (**e**) miR-183, (**f**) miR-200a, (**g**) miR-200b, (**h**) miR-200c, and (**i**) miR-429. The mean miRNA expression levels in the breast cancer tissues are significantly higher than that in pair-matched non-cancerous breast tissues. Data are reported as mean ± SD. * *p* < 0.05, ** *p* < 0.01, *** *p* < 0.001.

**Figure 4:**
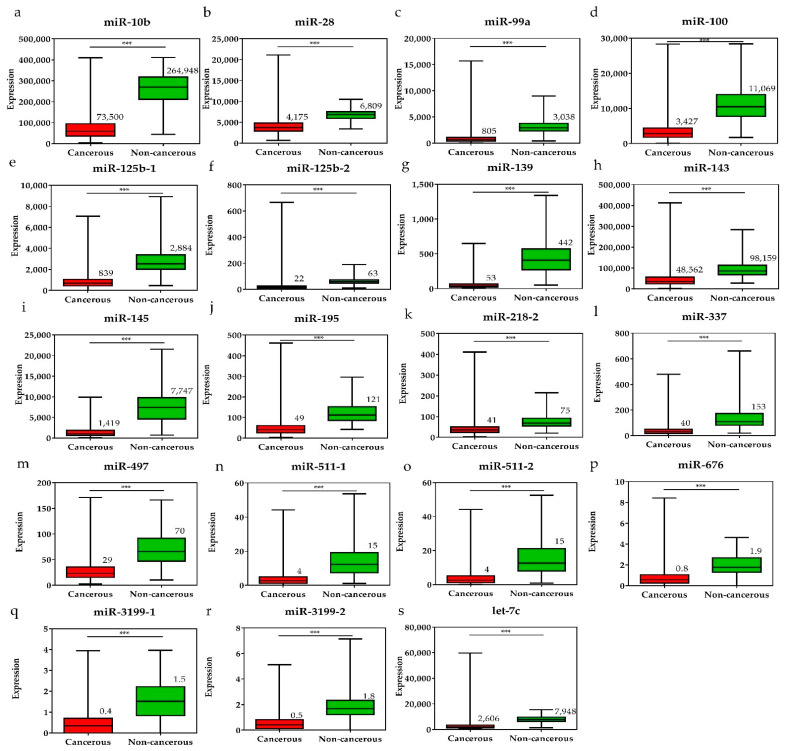
The expression levels of downregulated miRNAs in breast cancer tissues and the pair-matched non-cancerous tissues. The miRNAs downregulated in breast cancer tissues were (**a**) miR-10, (**b**) miR-28, (**c**) miR-99a, (**d**) miR-100, (**e**) miR-125b-1, (**f**) miR-125b-2, (**g**) miR-139, (**h**) miR-143, (**i**) miR-145, (**j**) miR-195, (**k**) miR-218-1, (**l**) miR-337, (**m**) miR-497, (**n**) miR-511-1, (**o**) miR-511-2, (**p**) miR-676, (**q**) miR-3199-1, (**r**) miR-3199-2, and (**s**) let-7c. The means of the miRNA expression levels in the breast cancer tissues are significantly lower than that in pair-matched non-cancerous breast tissues. Data are reported as mean ± SD. * *p* < 0.05, ** *p* < 0.01, *** *p* < 0.001.

To investigate the diagnostic value of the 28 selected miRNAs, the expression levels of these miRNAs were tested, and were found to be significantly higher or lower in breast cancer tissues than in adjacent non-cancerous tissues (*p* < 0.001, Figure 3 and Figure 4). The diagnostic performance of the 28 DE miRNAs was determined using ROC curve analysis. The AUCs of the 28 DE miRNAs are listed in Table 2. The AUCs for the top five miRNAs exceeded 0.97: miR-139 (0.99, 95% CI = 0.98–1.00), miR-21 (0.98, 95% CI = 0.97–0.99), miR-96 (0.97, 95% CI = 0.96–0.99), miR-183 (0.97, 95% CI = 0.96–0.99), and miR-10b (0.97, 95% CI = 0.96–0.99), indicating good diagnostic performance in breast cancer patients (*p* < 0.0001). To confirm the diagnostic value of the DE miRNAs, the combination of the top five miRNAs was analyzed. It showed improved sensitivity (96.95%, 95% CI = 95.46–98.06) and specificity (100%, 95% CI = 95.80–100.00) (Table 2).

**Figure 5:**
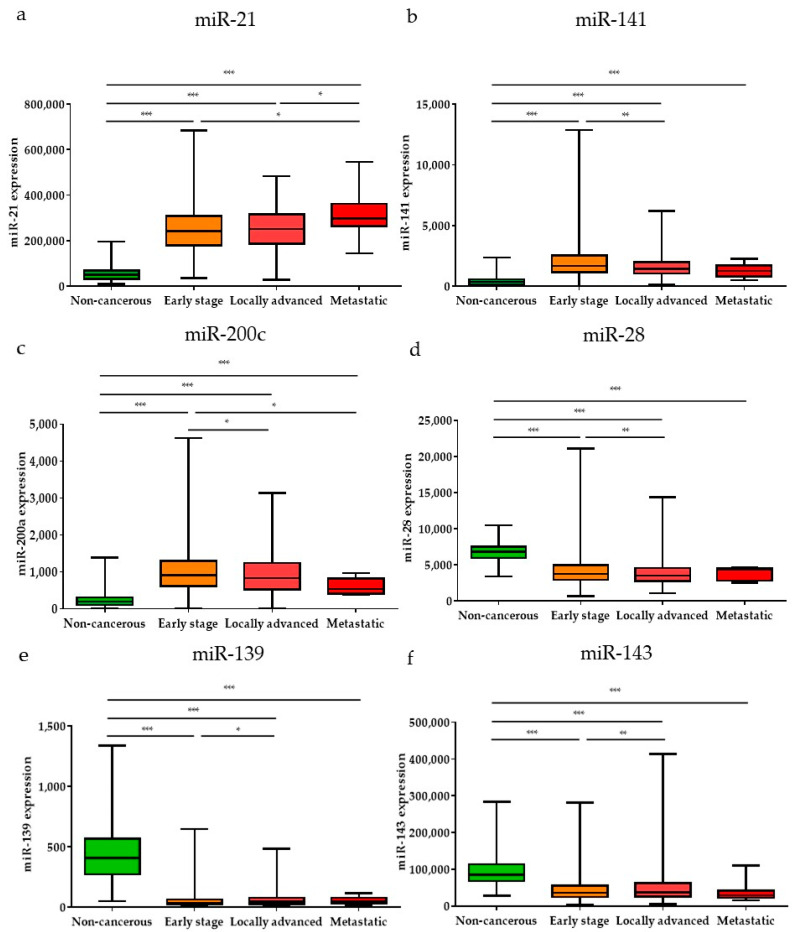
The significant DE miRNAs according to cancer stage. Among the upregulated miRNAs, (**a**) miR-21, (**b**) miR-141, and (**c**) miR-200c were significantly different in early, locally advanced, and metastatic stages. Among the downregulated miRNAs, (**d**) miR-28, (**e**) miR-139, and (**f**) miR-143 were significantly different in early, locally advanced, and metastatic stages. Data are reported as mean ± SD. * *p* < 0.05, ** *p* < 0.01, *** *p* < 0.001.

**Figure 6:**
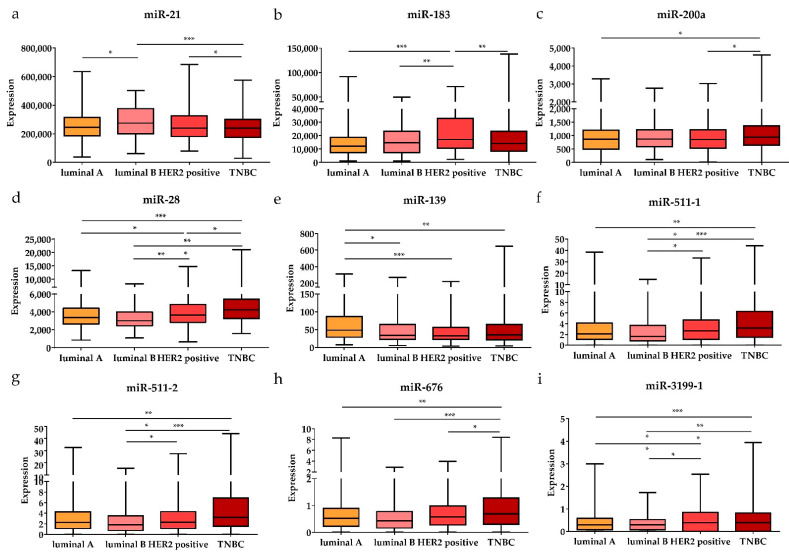
The significant DE miRNAs according to breast cancer subtypes. The upregulated (**a**) miR-21, (**b**) miR-183, and (**c**) miR-200a were significantly associated with different subtypes (luminal A, luminal B, HER2-positive, and TNBC). The downregulated (**d**) miR-28, (**e**) miR-139, (**f**) miR-511-1, (**g**) miR-511-2, (**h**) miR-676, and (**i**) miR-3199-1 were significantly associated with different subtypes. Data are reported as mean ± SD. * *p* < 0.05, ** *p* < 0.01, *** *p* < 0.001.

Subsequently, the expression levels of the DE miRNAs were investigated according to the cancer stage (Figure 5). Among the DE miRNAs, the expression level of miR-21 was significantly higher in the metastatic stage (stage 1) and locally advanced (stage 3) stage compared to the early stages (stages 1 and 2) (*p* < 0.05); the expression levels of miR-141 and miR-200c were significantly higher in the early stages than in the locally advanced stage (*p* < 0.01 and *p* < 0.05, respectively). The expression levels of miR-28, miR-139, and miR-143 were significantly lower in the early stages than in the locally advanced stage (*p* < 0.001, *p* < 0.05, and *p* < 0.01, respectively). Furthermore, the expression levels of the DE miRNAs were also analyzed by breast cancer subtypes (Figure 6). Among the nine upregulated miRNAs, the expression level of miR-21 was significantly higher in luminal B than in luminal A and TNBC (*p* < 0.05 and *p* < 0.001, respectively). The expression of miR-183 was significantly upregulated in HER2-positive patients compared to other subtypes (luminal A, luminal B, and TNBC) (*p* < 0.001, *p* < 0.01, and *p* < 0.01, respectively). The miR-200a expression level in TNBC was higher than that in luminal A and HER2-positive subtypes (*p* < 0.05 and *p* < 0.05, respectively). For the 19 downregulated miRNAs, the expression levels of miR-28 and miR-3199-1 in luminal A and luminal B were significantly downregulated compared to HER2-positive and TNBC. The expression level of miR-511-1 and miR-511-2 in luminal B were downregulated compared to HER2-positive and TNBC (*p* < 0.05 and *p* < 0.001 for both miR-511-1 and miR-511-2). The MiR-139 expression level in luminal A was significantly higher than that in luminal B, HER2, and TNBC (*p* < 0.05, *p* < 0.001, and *p* < 0.01, respectively); miR-676 expression in TNBC was significantly higher than that in luminal A, luminal B, and HER2-positive (*p* < 0.01, *p* < 0.001, and *p* < 0.05, respectively).

### 3.4. Survival for Selected miRNAs

To investigate the prognostic value of the 28 selected miRNAs, survival data with miRNA expressions were analyzed using miRpower (Figure S1). The results showed that high expression of miR-21, miR-141, and miR-200c was significantly associated with poor survival (*p* = 0.01, *p* < 0.001, and *p* < 0.001, respectively). The low expression of the ten downregulated miRNAs, namely, miR-10b, miR-99a, miR-100, miR-125b, miR-143, miR-145, miR-195, miR-218, miR-497, and let-7c, showed a significant correlation with poor survival.

### 3.5. Identification of Downstream Target Genes of miRNAs in Breast Cancer

**Figure 7:**
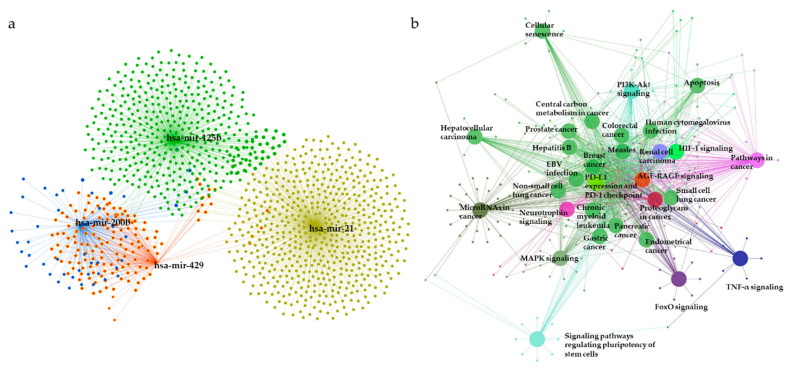
The miRNA–mRNA interaction network analysis and enriched KEGG pathways for target genes. (**a**) A significant miRNA target gene network is constructed by submitting the 28 selected miRNAs to the miRNet database. (**b**) The network represents KEGG pathways for target genes of the most significant miRNAs. Representations are generated by ClueGO for functionally grouped networks of enriched KEGG pathways. The parameters of GlueGo were set as follows: GO term fusion selected; only display pathways with *p* < 0.001 with Bonferroni step-down analysis; and kappa score of 0.4. MAPK: Mitogen-activated protein kinase; HIF-1: Hypoxia-inducible factor; PD-L1: Programmed cell death-ligand 1; PD-1: Programmed cell death-1; PI3K: Phosphatidylinositol 3-kinase; TNF: Tumor necrosis factor; FoxO: Forkhead box protein O; EBV: Epstein-barr virus.

To elucidate the underlying biological functions of miRNAs via negative regulation of the expression of downstream target genes, miRNet was used to predict the target genes of the 28 DE miRNAs. As shown in Figure 7a, a total of 1381 predicted target genes of miR-21 (yellow), miR-125b (green), miR-200b (blue), and miR-429 (red) were obtained. The list of predicted target genes is shown in Table S2.

Next, the predicted target genes were analyzed using KEGG pathway enrichment analysis with the ClueGO plug-in of Cytoscape (kappa score = 0.4, *p* < 0.001 with Bonferroni step-down analysis) (Figure 7b, Table 3). The relationships between pathways were observed, and predicted target genes were found to be enriched in mitogen-activated protein kinase (MAPK) signaling, hypoxia-induced factor-1 (HIF-1) signaling, central carbon metabolism in cancer, programmed cell death-ligand 1 (PD-L1) expression, and programmed cell death-1 (PD-1) checkpoint pathway in cancer, phosphatidylinositol 3-kinase (PI3K)-Akt signaling, apoptosis, signaling pathways regulating pluripotency of stem cells, tumor necrosis factor (TNF) signaling, pathways in cancer, and microRNAs in cancer pathways. KEGG pathways for the predicted targets are summarized in Table S3.

## 4. Discussion

Breast cancer is one of the most commonly diagnosed cancers and causes of significant cancer-mediated deaths in women worldwide [27]. Moreover, despite the constant development of diagnostic approaches for cancer, early diagnosis of breast cancer and improvement in survival remain difficult. It has been shown that various imaging approaches, such as mammography, magnetic resonance imaging, positron emission tomography, computed tomography, and single-photon emission computed tomography, can be used for the diagnosis and monitoring of breast cancer patients in various stages [28,29,30]. Currently, numerous studies on new diagnostic approaches for breast cancer using circulating tumor cells, circulating tumor DNA, exosomes, and microRNAs are underway [31,32,33,34].

The miRNAs, a group of small, single-stranded, non-coding RNA molecules, are frequently dysregulated in cancers, including breast cancer [35]. Recent studies have found that specific miRNAs are associated with breast cancer [36,37]. Studies on the clinical applications of miRNAs, such as in diagnosis, prognosis, and therapeutic strategies for cancer, including breast cancer, are also gaining prominence [21]. Here, a systematic analysis of miRNA expression profiles from TCGA was performed to identify potential miRNAs for the diagnosis of breast cancer. First, 28 DE miRNAs were screened for expression in breast cancer tissues compared to adjacent non-cancerous tissues, identifying nine upregulated and 19 downregulated miRNAs. Of these, miR-21 and miR-139 were found to be the most significantly upregulated and downregulated miRNAs, respectively, in breast cancer tissues. Previous studies have shown that miR-21 overexpression in breast cancer is associated with cell proliferation, progression, metastasis, and poor prognosis [38,39]. It has also been reported that miR-21 promotes invasion and cell proliferation by targeting programmed cell death 4 (PDCD4) [38]; miR-139 has been reported to act as a tumor suppressor in several cancer types, such as prostate cancer, endometrial cancer, and breast cancer [40,41,42]. In addition to miR-21 and miR-139, selected DE miRNAs have also been confirmed to function as one of the major components in cancer biology by other groups. The identified DE miRNAs have been studied for their tumor-suppressive or oncogenic functions, but their diagnostic potential in clinical settings has not been fully elucidated.

Therefore, to evaluate the selected DE miRNAs as diagnostic tools for breast cancer, their performance characteristics of sensitivity, specificity, and AUC were analyzed. The results showed a sensitivity of 97%–76% and a specificity of 98%–80%. The AUC values ranged from 0.99 (95% CI = 0.98–1.00) to 0.83 (95% CI = 0.77–0.88) (*p* < 0.0001). These values are higher than the previously reported sensitivity of 67%–95% and specificity of >95% using the current standard diagnostic tools, such as mammography [43,44]. Several studies have investigated miRNAs for the diagnosis of breast cancer. Hastings et al. reported that the expression levels of miR-148b, miR-376c, and miR-409-3p were upregulated in benign breast tissues compared to those in breast cancer tissues [45]. Additionally, Cookson et al. showed that upregulation of miR-16, miR-21, and miR-451 and downregulation of miR-145 in the plasma of breast cancer patients serves as a screening biomarker [8]. Moreover, the miRNA profile analysis of miR-1, miR-92a, miR-133a, and miR-133b in breast cancer suggested their potential diagnostic performance with high AUC values (0.90 to 0.91) [46]. Taken together, the clinical relevance of the 28 selected DE miRNAs was comparable to that of the other miRNAs.

Classification into molecular subtypes based on the presence or absence of three receptors, the ER, PR, and HER2, is considered the gold standard for diagnosis and prognosis, providing essential information for accelerating therapeutic decisions (chemotherapy, hormone therapy, and anti-HER2 therapy) [47,48]. Among the DE miRNAs, the results revealed an upregulation of miR-200a in TNBC, miR-183 in HER2-positive, and miR-21 in luminal B. Moreover, we observed a downregulation of miR-28, miR-3199-1, miR-511-1, and miR-511-2 in luminal B. Several studies identified subtype-specific dysregulated miRNAs in breast cancer. Shin et al. suggested that downregulation of miR-16, miR-21, miR-199a, miR-185, and miR-143 and upregulation of miR-92a-3p, miR-23b-3p, and miR-343-3p could be used to discriminate between TNBC and non-TNBC [49]. Other studies revealed an upregulation of miR-373 in ER-positive [50], miR-342 in luminal B [51], and miR-18b, miR-103, miR-107, and miR-652 in TNBC [52]. In this context, the possible role has been suggested of miRNAs’ specific response to breast cancer subtype, emerging as a potential diagnostic marker.

Several studies have also reported that the expression levels of some miRNAs (miR-21, miR-23b, and miR-24-3p) are associated with poor prognosis in breast cancer [53,54,55]. In this study, the results showed that high expression levels of miR-21, miR-141, and miR-200c were more closely related to poor survival. Furthermore, low expression levels of miR-10b, miR-99a, miR-100, miR-125b, miR-143, miR-145, miR-195, miR-218, miR-497, and let-7c had a significantly shorter survival time than those with high expression.

To establish the functional features of the DE miRNAs, miRNet was used for predicting target mRNAs, and pathway analysis for the predicted targets using KEGG were performed. Significant target genes for miR-21, miR-125b, miR-200b, and miR-429 were identified. The identified target genes are involved in breast cancer, PD-L1 expression and PD-1 checkpoint pathway in cancer, MAPK signaling, apoptosis, and TNF pathways. In particular, PD-1/PD-L1 is expressed on the surface of immune cells, such as T-cells, B-cells, and natural killer T cells, which function as immune checkpoint inhibitors [56,57,58,59]. PD-L1 in cancer cells binds to PD-1 present in T cells, inhibiting T cell function [60]. PD-L1 expression is associated with the occurrence of larger tumor size, high grade, estrogen receptor-negative, progesterone receptor-negative, and HER2-positive breast cancer [61]. PD-L1 is also expressed in 20% of TNBCs [62]. Recent studies have attempted to block the PD-1/PD-L1 pathway to ensure stronger tumor regression in cellular immunotherapies [63,64,65,66,67]. Thus, these results could improve the course of further research on immunotherapeutic strategies.

## 5. Conclusions

In conclusion, this study provides a comprehensive analysis of DE miRNAs and their potential targets and diagnostic performance in breast cancer. They may serve as promising diagnostic biomarkers. Additionally, these dysregulated miRNAs should be further investigated using tissue samples and blood samples collected from multiple centers at various stages and subtypes, such as luminal A, luminal B, HER2, and basal breast cancer. Further studies are also needed for validation for DE miRNA targets and prognostic values considering survival days, lymph node, surgical type, and adjuvant treatment.

## Figures and Tables

**Table 1:** Clinical characteristics of breast cancer patients.

Characteristics	Breast Cancer (*n* = 755)
Female, *n* (%)	746 (98.8)
Age (y, mean ± SD)	58.42 ± 13.11
Race, *n* (%)	
Asian	56 (7.42)
Black or African American	156 (20.66)
White	537 (71.13)
Unknown	6 (0.79)
Pathological stage	
Early (stage I and II)	567 (75.10)
Locally advanced (stage III)	171 (22.65)
Metastatic (stage IV)	9 (1.19)
Unknown	8 (1.06)
Molecular subtype	
Luminal A	208 (27.55)
Luminal B	74 (9.80)
HER2 positive	116 (15.36)
TNBC	357 (47.28)

**Table 2:** Comparison of the diagnostic utility of the 28 differentially expressed miRNAs in breast cancer.

miRNAs	Expression Levels		AUC	Cutoff	Sensitivity	Specificity	*p*-Value
	Cancerous	Non-Cancerous					
miR-139	53.78 ± 51.39	442.40 ± 238.40	0.99 (0.98–1.00)	<142.40	94.44% (92.55–95.96)	97.67% (91.85–9.72)	<0.0001
miR-21	249857 ± 98314	54433 ± 32501	0.98 (0.97–0.99)	>89285	96.82% (95.31–97.95)	93.02% (85.43–97.40)	<0.0001
miR-96	42.21 ± 33.52	4.48 ± 4.54	0.97 (0.96–0.99)	>9.49	92.32% (90.18–94.12)	90.70% (82.49–95.90)	<0.0001
miR-183	17528 ± 13739	2123 ± 1687	0.97 (0.96–0.99)	>4366	93.11% (91.07–94.81)	95.35% (88.52–98.72)	<0.0001
miR-10b	73500 ± 52246	264948 ± 71301	0.97 (0.96–0.99)	<155979	93.11% (91.07–94.81)	94.19% (86.95–98.09)	<0.0001
miR-145	1419 ± 1189	7747 ± 4123	0.97 (0.95–0.99)	<2992	92.05% (89.89–93.88)	93.02% (85.43–97.40)	<0.0001
miR-99a	805.30 ± 835.00	3038 ± 1278	0.96 (0.94–0.98)	<1506	90.86% (88.58–92.82)	93.02% (85.43–97.40)	<0.0001
miR-182	46895 ± 34488	8578 ± 5819	0.95 (0.94–0.97)	>14180	90.73% (88.43–92.70)	90.70% (82.49–95.90)	<0.0001
let-7c	2606 ± 2826	7948 ± 2638	0.95 (0.92–0.97)	<4359	87.28% (84.70–89.58)	93.02% (85.43–97.40)	<0.0001
miR-141	1939 ± 1314	424.60 ± 404.00	0.94 (0.92–0.97)	>870.90	86.23% (83.56–88.60)	90.70% (82.49–95.90)	<0.0001
miR-125b-1	839.10 ± 656.90	2884 ± 1483	0.94 (0.92–0.97)	<1322	86.49% (83.84–88.85)	93.02% (85.43–97.40)	<0.0001
miR-125b-2	22.13 ± 29.51	63.66 ± 28.27	0.94 (0.92–0.96)	<35.60	86.36% (83.70–88.73)	90.70% (82.49–95.90)	<0.0001
miR-100	3427 ± 2602	11069 ± 4621	0.94 (0.91–0.97)	<5597	86.49% (83.84–88.85)	90.70% (82.49–95.90)	<0.0001
miR-200a	1016 ± 626.40	225.6 ± 203.0	0.94 (0.91–0.96)	>370.00	90.07% (87.71–92.11)	84.88% (75.54–91.70)	<0.0001
miR-429	123.30 ± 98.28	20.86 ± 21.94	0.93 (0.91–0.96)	>32.56	90.33% (88.00–92.34)	86.05% (76.89–92.58)	<0.0001
miR-195	49.86 ± 40.15	121.5 ± 46.99	0.92 (0.90–0.94)	<67.69	80.93% (77.94–83.67)	93.02% (85.43–97.40)	<0.0001
miR-337	40.46 ± 41.63	153.30 ± 117.30	0.92 (0.89–0.95)	<67.89	85.96% (83.28–88.36)	86.05% (76.89–92.58)	<0.0001
miR-200c	12257 ± 7356	4191 ± 3181	0.90 (0.87–0.93)	>6359	82.38% (79.48–85.04)	84.88% (75.54–91.70)	<0.0001
miR-200b	739.40 ± 491.60	224.3 ± 168.2	0.89 (0.86–0.93)	>334.00	83.97% (81.16–86.52)	82.56% (72.87–89.90)	<0.0001
miR-3119-2	0.56 ± 0.59	1.84 ± 1.04	0.89 (0.85–0.93)	<1.03	85.03% (82.29–87.50)	87.21% (78.27–93.44)	<0.0001
miR-511-2	4.31 ± 5.41	15.64 ± 10.54	0.89 (0.85–0.92)	<6.64	81.72% (78.78–84.42)	86.05% (76.89–92.58)	<0.0001
miR-497	29.01 ± 22.06	70.12 ± 32.43	0.89 (0.85–0.92)	<41.53	81.19% (78.22–83.92)	84.88% (75.54–91.70)	<0.0001
miR-28	4175 ± 2047	6809 ± 1514	0.88 (0.85–0.91)	<5414	81.46% (78.50–84.17)	84.88% (75.54–91.70)	<0.0001
miR-511-1	4.33 ± 5.58	15.05 ± 10.74	0.88 (0.84–0.91)	<6.70	81.99% (79.06–84.66)	80.23% (70.25–88.04)	<0.0001
miR-143	48562 ± 42798	98159 ± 46495	0.86 (0.83–0.89)	<64538	80.40% (77.38–83.17)	82.56% (72.87–89.90)	<0.0001
miR-218-2	41.07 ± 30.26	75.87 ± 34.19	0.85 (0.81–0.89)	<51.24	76.03% (72.82–79.03)	82.56% (72.87–89.90)	<0.0001
miR-676	0.82 ± 0.94	1.96 ± 1.03	0.84 (0.80–0.88)	<1.11	77.09% (73.92–80.04)	80.23% (70.25–88.04)	<0.0001
miR-3199-1	0.47 ± 0.53	1.52 ± 0.95	0.83 (0.77–0.88)	<0.74	77.35% (74.20–80.29)	80.23% (70.25–88.04)	<0.0001
**Combination of top 5 miRNAs**					**Sensitivity**	**Specificity**	***p*** ** value**
miR-139 + mir-21 + miR-96 + miR-183 + miR-10b					96.95% (95.46–98.06)	100.00% (95.80–100.00)	<0.0001

**Table 3:** List of gene ontology terms for predicted targets of differentially expressed miRNAs.

GO ID	GO Terms	No. of Genes	*p*-Value
KEGG:04010	MAPK signaling pathway	53	<0.001
KEGG:04066	HIF-1 signaling pathway	32	<0.001
KEGG:05230	Central carbon metabolism in cancer	20	<0.001
KEGG:05235	PD-L1 expression and PD-1 checkpoint pathway in cancer	24	<0.001
KEGG:04151	PI3K-Akt signaling pathway	56	<0.001
KEGG:04210	Apoptosis	30	<0.001
KEGG:04550	Signaling pathways regulating pluripotency of stem cells	32	<0.001
KEGG:04668	TNF signaling pathway	27	<0.001
KEGG:04722	Neurotrophin signaling pathway	32	<0.001
KEGG:04933	AGE-RACE signaling pathway in diabetic complications	28	<0.001
KEGG:05200	Pathways in cancer	96	<0.001
KEGG:05205	Proteoglycans in cancer	48	<0.001
KEGG:05206	microRNAs in cancer	76	<0.001
KEGG:05211	Renal cell carcinoma	20	<0.001
KEGG:04068	FoxO signaling pathway	36	<0.001
KEGG:05215	Prostate cancer	35	<0.001
KEGG:05161	Hepatitis B	45	<0.001
KEGG:05162	Measles	32	<0.001
KEGG:05169	EBV infection	40	<0.001
KEGG:05220	Chronic myeloid leukemia	27	<0.001
KEGG:05222	Small cell lung cancer	26	<0.001
KEGG:04218	Cellular senescence	36	<0.001
KEGG:05163	Human cytomegalovirus infection	42	<0.001
KEGG:05210	Colorectal cancer	28	<0.001
KEGG:05212	Pancreatic cancer	27	<0.001
KEGG:05220	Chronic myeloid leukemia	27	<0.001
KEGG:05223	Non-small cell lung cancer	21	<0.001
KEGG:05224	Breast cancer	37	<0.001
KEGG:05225	Hepatocellular carcinoma	41	<0.001
KEGG:05226	Gastric canner	39	<0.001

GO: gene ontology; KEGG: Kyoto Encyclopedia of Genes and Genomes.

## Supplementary Materials

The following are available online at https://www.mdpi.com/2075-4418/11/1/107/s1; Figure S1: Kaplan–Meier survival for low DE miRNAs versus high DE miRNAs expression level, Table S1: Clinical information from TCGA, Table S2: List of predicted target genes, Table S3: KEGG pathways for predicted targets.
